# Power Restoration Optimization Strategy for Active Distribution Networks Using Improved Genetic Algorithm

**DOI:** 10.3390/biomimetics10090618

**Published:** 2025-09-14

**Authors:** Pengpeng Lyu, Qiangsheng Bu, Yu Liu, Jiangping Jing, Jinfeng Hu, Lei Su, Yundi Chu

**Affiliations:** 1 State Grid Jiangsu Electric Power Co., Ltd. Research Institute, Nanjing 211103, China; lvpp@js.sgcc.com.cn (P.L.); boqs@js.sgcc.com.cn (Q.B.); 2 College of Artificial Intelligence and Automation, Hohai University, Nanjing 210098, China; 231322020002@hhu.edu.cn; 3 State Grid Jiangsu Electric Power Co., Ltd., Nanjing 210024, China; jingjp1@js.sgcc.com.cn (J.J.); jfhu@js.sgcc.com.cn (J.H.); yxsulei@js.sgcc.com.cn (L.S.)

**Keywords:** power restoration duration, genetic algorithm, critical load recovery, distributed resources, microgrid, island partitioning

## Abstract

During feeder outages in the distribution network, localized power restoration using distribution resources (e.g., PVs) can ensure supply to critical loads and mitigate adverse impacts, especially when main grid support is unavailable. This study presents a power restoration strategy aiming at maximizing the restoration duration of critical loads to ensure their prioritized recovery, thereby significantly improving power system reliability. The methodology begins with load enumeration via breadth-first search (BFS) and utilizes a long short-term memory (LSTM) neural network to predict microgrid generation output. Then, an adaptive multipoint crossover genetic solving algorithm (AMCGA) is proposed, which can dynamically adjust crossover and mutation rates, enabling rapid convergence and requiring fewer parameters, thus optimizing island partitioning to prioritize critical load demands. Experimental results show that AMCGA improves convergence speed by 42.5% over the traditional genetic algorithm, resulting in longer restoration durations. Compared with other strategies that do not prioritize critical load recovery, the proposed strategy has shown superior performance in enhancing critical load restoration, optimizing island partitioning, and reducing recovery fluctuations, thereby confirming the strategy’s effectiveness in maximizing restoration and improving stability.

## 1. Introduction

With the increasing integration of distributed resources and advanced control technologies, the complexity of distribution networks has significantly increased. During feeder outages, where main grid support is unavailable, localized restoration using distribution resources can ensure continued power supply to critical loads. This highlights the need for innovative power restoration strategies [1]. Effective restoration strategies hinge on precise, real-time information awareness under uncertainties such as distributed energy resources fluctuations, load variations, and topological reconfigurations. The deployment of Phasor Measurement Units (PMUs) for accurate real-time monitoring is thus fundamental to preventing system failures and blackouts, providing critical technical support for applications like local power restoration [2,3,4]. The integration of microgrids enables localized power restoration during transmission failures and distribution blackouts, allowing power to be supplied to local areas without disconnecting from the main grid [5,6]. Therefore, the development of efficient restoration strategies is essential to ensure a reliable power supply to critical loads and enhance the overall system resilience.

Optimization methods, particularly genetic algorithms, have been widely utilized in developing power restoration strategies for distribution networks [7,8,9,10]. This study, inspired by the concepts presented in [11], employs a loop-based integer encoding scheme to efficiently solve the complex topological optimization problem. Furthermore, traditional distribution network planning with distributed PV has relied on centralized models, which become exceedingly complex when considering numerous decision variables. Our loop-based methodology can decompose the large-scale optimization problem into more manageable subproblems. This zonal approach makes the optimization feasible even when the planning model must account for complex factors such as the investment in PV facilities [12]. For instance, a genetic algorithm that encodes parameters related to nodes and branches was employed in [13] to tackle power restoration challenges, while an approach integrating loop establishment and population processing was proposed in [11] to effectively address the power restoration issue. However, genetic algorithms face limitations in parameter adjustment and exhibit restricted diversity and global search performance due to their singular crossover point design, resulting in poor convergence [14]. Thus, there is a pressing need for a novel genetic algorithm (GA) to enhance the effectiveness and efficiency of power restoration strategies.

In addition, extensive research has been conducted both domestically and internationally on the application of microgrids with distributed generation (DG) resources in the operation and fault reconfiguration of distribution networks. Efforts have focused on optimizing the allocation of distributed energy resources to enhance the efficiency and reliability of distribution networks [15,16,17]. Moreover, microgrids have played a pivotal role in developing multi-timescale power restoration strategies [18,19]. Despite this progress, existing studies often overlook the time-varying nature of DG output, limiting the adaptability and flexibility of current restoration strategies [20]. In addition, microgrid partitioning has become increasingly important for restoring power supplies in distribution networks [21,22]. The work in [23] examined the recovery of loads near the microgrid, proposing a novel method to ensure the restoration of specific loads and highlighting the practical value of microgrids in providing localized power for restoration. However, existing research frequently overlooks the prioritization of critical loads during the restoration process and fails to fully explore the sustained application of microgrid operation modes. Furthermore, ensuring a continuous power supply to critical loads is essential for both system reliability and the uninterrupted operation of key infrastructure [24,25,26,27,28]. Yet, most current studies neglect the temporal variability of DG output and the continuity of power supply, failing to adequately address the maximization of the critical load power supply duration.

To tackle the challenge of restoring power mentioned before, this paper presents a power restoration strategy that maximizes restoration duration for critical loads. The key contributions of this study are listed as follows:

(1) A power restoration model is developed with the objective of maximizing the restoration duration of critical loads, thereby ensuring their continuous supply across various operating conditions in complex scenarios involving total blackouts in both the main grid and distribution network.
(2) An adaptive multi-point crossover genetic algorithm (AMCGA) is introduced to enhance search efficiency and global search capability by dynamically adjusting crossover operations, thus ensuring the reliability and optimality of the power restoration strategy.
(3) The integration of microgrid power supply partitioning strategies optimizes the prioritization of diverse load types, thereby facilitating the preferential restoration of critical loads and further enhancing the restoration duration of essential services.

## 2. Island Division and Uninterrupted Power Supply Recovery Path

Effective restoration strategies hinge on precise, real-time information awareness, especially under the uncertainties introduced by distributed resources and dynamic topologies. The deployment of PMUs and Intelligent Electronic Devices (IEDs) is fundamental for wide-area monitoring and robust state estimation, providing the necessary technical support for advanced applications like power restoration [29]. This study, therefore, presupposes an active distribution network equipped with such an advanced metering infrastructure. The high-fidelity, time-synchronized data provided by this system is considered a direct input for our model, enabling the subsequent optimization algorithms to operate on an accurate and reliable representation of the network’s state.

In the event of a fault or large-scale blackout, the real-time, high-precision grid status provided by PMUs enables the effective collaboration of island division and uninterrupted power supply recovery paths to restore power. This section briefly describes the relationship between island division and uninterrupted power supply recovery paths in fault recovery, as well as how island division is used to determine uninterrupted supply recovery paths.

### 2.1. Island Division

In the context of power system fault scenarios, partitioning the distribution network into autonomous operational regions is crucial for ensuring the continuous supply of electricity to critical loads. This partitioning process prioritizes regions based on factors such as critical load demand, the capacity of distributed generation (DG) systems, and operational constraints of the grid. To enhance the reliability of the division, LSTM neural networks are employed to analyze historical generation data and real-time system conditions, delivering accurate forecasts of microgrid output. These predictive models facilitate a comprehensive assessment of each region’s ability to sustain critical loads in the aftermath of system disturbances. The identified operational regions serve as the foundation for subsequent recovery path planning, ensuring that areas with stable local generation and high-priority loads are restored first, thereby optimizing the fault recovery process.

### 2.2. Uninterrupted Power Supply Recovery Path Based on Loop

The design of the uninterrupted power supply recovery path, based on the fundamental loop structure of the distribution network, emphasizes the preservation of critical load continuity during fault conditions [11]. This design strategy incorporates a breadth-first search (BFS) algorithm to construct a minimum spanning tree, followed by the application of shortest path optimization techniques to determine efficient restoration routes. Using the standard LSTM model from [30], we forecasted the annual output of a photovoltaic microgrid in Jiangsu, China. The resulting microgrid output predictions play a crucial role in the path selection decision-making process, providing accurate predictions of generation availability along potential recovery routes. These forecasts enable the system to prioritize restoration sequences by integrating both topological efficiency and actual output availability. Through the systematic restoration of self-sustaining islands containing critical loads, the recovery strategy enhances overall system efficiency, minimizing both the restoration time and the extent of system disruption.

## 3. Power Restoration Model Considering the Maximization of Critical Load Recovery Duration

In the case of widespread power outages in the distribution network caused by transmission grid faults, it is crucial to develop an effective power restoration strategy that prioritizes the restoration of critical loads and ensures their continuous power supply. For distribution networks with grid-connected microgrids, the microgrid can provide necessary power to specific loads without disconnecting from the main grid. Given the volatility of microgrid generation, the restoration strategy must be adapted dynamically based on the predicted output of the microgrid to prioritize the restoration of critical loads and maximize recovery duration in practical power systems. The model for this strategy is characterized by competing objectives, with the primary goal of maximizing the total weighted restoration duration for critical loads during a main grid outage. This primary objective is in direct competition with secondary goals, such as maximizing the restoration duration of non-critical loads. To prioritize and ensure supply to critical loads, the model may opt to sacrifice non-critical loads. These conflicting objectives collectively constitute a complex model that must be optimized subject to strict physical constraints.

Within the context of a PMU-enabled grid, prioritizing load restoration is critical for mitigating the societal impact of power outages. This model classifies loads based on their importance to essential services to ensure a structured and effective recovery process. Primary Loads (e.g., command centers), Secondary Loads (e.g., hospitals, traffic systems), and Tertiary Loads are assigned distinct weights. This classification ensures that our subsequent optimization algorithm prioritizes maximizing the supply duration for these vital loads.

### 3.1. Objective Function

The power restoration model aims to maximize the restoration duration of critical loads, ensuring public safety and the continuity of essential services during outages. In extreme scenarios, prioritizing critical load restoration enhances recovery stability by ensuring the timely restoration of essential services. The objective function is given as follows:

(1) $$\max f=\sum_{i=0}^{n}\sum_{j=1}^{m}\omega_{j}\cdot t_{i\_j}$$

where f represents the objective function, indicating the restoration duration for all loads in the distribution network during the period of main grid failure; n refers to the number of time intervals defined within the expected main grid restoration time; m denotes the total number of loads within the power system topology; $\omega_{j}$ is the weight associated with a single load; $t_{i\_j}$ is the load restoration time corresponding to node j during time interval i.

### 3.2. Model Constraints

The constraints that must be satisfied are listed as follows:

(1) Nodal Power Balance Constraint:
  (2) $$\left\{\begin{array}{l} P_{Gi}+P_{DGi}-P_{Li}=U_{i}\sum_{j=1}^{N}U_{j}(G_{ij}\cos\theta_{ij}+B_{ij}\sin\theta_{ij})\\ Q_{Gi}+Q_{DGi}-Q_{Li}=U_{i}\sum_{j=1}^{N}U_{j}(G_{ij}\cos\theta_{ij}+B_{ij}\sin\theta_{ij}) \end{array}\right.$$
  where $P_{Gi}$ and $Q_{Gi}$ represent the active and reactive power injected at node i, respectively. $P_{DGi}$ and $Q_{DGi}$ denote the active and reactive power injected by the DG at node i. $P_{Li}$ and $Q_{Li}$ indicate the active and reactive loads at node i, respectively. $U_{i}$ and $U_{j}$ refer to the voltages at the starting and ending nodes of the branch, specifically nodes i and j. $G_{ij}$ and $B_{ij}$ represent the conductance and susceptance between nodes i and j, respectively. Finally, $\theta_{ij}$ indicates the phase difference between nodes i and j.
(2) Node Voltage Constraint:
  (3) $$U_{i}^{\min}\leq U_{i}\leq U_{i}^{\max}$$
  where $U_{i}^{\min}$ and $U_{i}^{\max}$ represent the minimum and maximum voltage levels at the i node, respectively.
(3) Branch Active Power Constraint:
  (4) $$|P_{i}|\leq P_{i}^{\max}$$
  where $P_{i}$ represents the active power of the branch i, and $P_{i}^{\max}$ denotes the allowable maximum power of the branch i.
(4) Microgrid Power Constraint:
  (5) $$P_{wi}^{\min}\leq P_{wi}\leq P_{wi}^{\max}$$
  where $P_{wi}^{\min}$ and $P_{wi}^{\max}$ represent the minimum and maximum generation power of the microgrid at the node i, respectively; $P_{wi}$ denotes the generation power of the microgrid at the i node.
(5) Island Power Constraint:
  (6) $$\sum_{j\in D_{i}}P_{j}\leq P_{wi}$$
  where $D_{i}$ represents the set of nodes within the i island; $P_{j}$ denotes the generation power at the j node within the island.

## 4. Adaptive Multipoint Crossover Genetic Algorithm for Power Restoration Strategy

The model in this strategy is characterized as a multi-objective, time-dependent nonlinear programming problem. The “multi-objective” nature is reflected in the need to balance competing objectives, such as maximizing the restoration duration of critical loads and maximizing the total amount of restored load. The “time-dependent” characteristic arises because the model relies on predictions of distributed generation output over a future time horizon post-fault to formulate the restoration strategy, making the decisions time-dependent. Finally, the “nonlinear” aspect is due to the inherent nonlinearity of physical constraints, such as power flow calculations. Given the high complexity of this problem, this paper employs a heuristic algorithm to find high-quality, near-optimal solutions within a reasonable computation time.

The genetic algorithm (GA), based on natural selection, is widely used for optimizing power restoration sequences. However, traditional GA faces limitations such as slow convergence speed and sensitivity to parameter settings, which hinder its effectiveness in complex power systems. Despite these challenges, GA still offers advantages in optimizing distribution network topological constraints. In the case of a power system blackout, this study develops AMCGA to optimize island division and uninterrupted power restoration paths. By integrating microgrid partitioning strategies, the approach facilitates the power restoration of isolated distribution areas and maximizes the restoration time of critical loads. This method enhances convergence speed and adaptability, providing a more efficient dynamic power restoration solution, thereby improving system recovery efficiency and stability.

### 4.1. Adaptive Multi-Point Crossover Genetic Algorithm

The proposed AMCGA is a population-based optimization algorithm. Each individual in the population represents a potential power restoration strategy, referred to as a “chromosome”, where each gene corresponds to a specific operation within the restoration strategy. AMCGA consists of five main phases: initialization, evaluation, selection, crossover, and mutation.

The core of AMCGA lies in its adaptive multi-point crossover mechanism, which dynamically adjusts crossover operations to enhance the diversity and adaptability of the population, thereby optimizing the restoration capability of the power system. During initialization, the power system topology is constructed, generating an ordered BFS pseudo-tree, and the population parameters are initialized. These parameters include the population size, the number of elite individuals (typically half of the total population), the initial crossover probability $P_{c1}$, the crossover rate adjustment coefficient $P_{c2}$, the initial mutation probability $P_{m}$, the crossover rate adjustment coefficient $P_{c2}$, the maximum number of iterations $I_{\max}$, and the current iteration number I. The mutation coefficient m decreases as the population size increases, while the crossover probability $P_{cross}$ and mutation probability $P_{mutation}$ are adjusted at each iteration based on the evolving population.

During the evaluation phase, AMCGA calculates the fitness of each chromosome after the population is updated in each generation. The fitness reflects the chromosome’s performance in power restoration tasks, such as restoration duration and system stability. This fitness is used to guide the selection of individuals in the next generation, promoting the propagation of high-quality solutions. The fitness calculation formula is designed as follows:

(7) $$fitness=\sum_{i=0}^{n}\sum_{j=1}^{m}\omega_{j}\cdot t_{i\_j}$$

In the selection phase, chromosomes are ranked based on fitness, and the top half are selected for further operations. A random number is generated for each chromosome; if it is smaller than the current crossover probability, the chromosome is marked for crossover; otherwise, it is not. The crossover probability is initially high to promote exploration of the solution space, and it gradually adjusts in later stages, with higher-fitness individuals having a greater chance of crossover, facilitating the propagation of advantageous traits. The crossover probability is calculated as follows:

(8) $$P_{cross}=P_{c1}+P_{c2}\cdot \frac{\left(I_{\max}-I\right)}{I_{\max}}$$

In the crossover phase, the adaptive multi-point crossover mechanism plays a key role. Initially, a higher crossover probability encourages exploration, preventing premature convergence to local optima. As iterations progress, the crossover probability adjusts based on individual fitness, and we refer to the fittest half of the population as elite individuals, with higher-fitness individuals more likely to crossover, transmitting beneficial features to offspring. The multi-point crossover operator exchanges traits at multiple positions of the parent chromosomes, increasing offspring diversity, preventing premature convergence, and enhancing the algorithm’s adaptability to various power restoration scenarios.

Following crossover, the offspring undergo fitness evaluation, and the resulting new individuals are processed in the mutation stage. In this phase, the mutation probability is also adjusted according to fitness feedback: lower-fitness chromosomes undergo more mutation, while higher-fitness chromosomes are less likely to undergo mutation. Specifically, if a random number between 0 and 1 is smaller than the mutation probability, a gene in the chromosome is replaced by a random value within its domain. The offspring and parent chromosomes are then merged into a new population, which undergoes the next iteration. The mutation probability is calculated as:

(9) $$P_{mutation}=P_{m}\cdot I_{\max}/\left(I_{\max}-m\cdot I\right)$$

Ultimately, AMCGA adapts the crossover and mutation probability based on the fitness feedback from the new generation, ensuring that the algorithm remains flexible across iterative stages. This dynamic adjustment process effectively integrates the evolutionary strengths of genetic algorithms with the specific requirements of power system recovery, enhancing both the efficiency and flexibility of the restoration strategy, and achieving optimal system reconfiguration.

### 4.2. Algorithmic Solution Process

We utilize PMUs for real-time monitoring of the power system. The core objective of our PMU placement strategy is to ensure measurement continuity for critical nodes, including important loads and microgrids. The resulting measurement redundancy, achieved through overlapping PMU coverage, guarantees that the restoration status of these assets remains accurately observable during network reconfiguration, even if a monitoring path is interrupted. This is crucial for reliably determining the optimal switching sequence.

**Figure 1:**
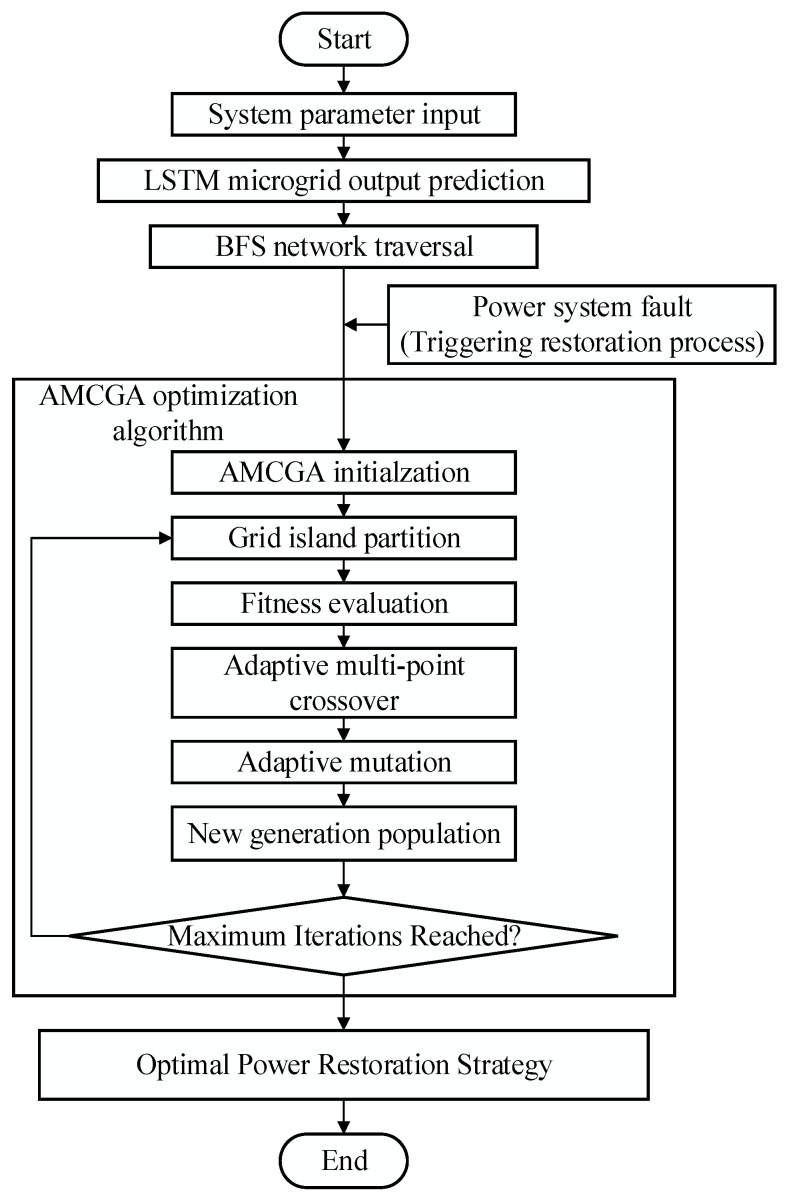
Adaptive multipoint crossover genetic algorithm for power restoration.

The power restoration process is shown in Figure 1.

First, real-time operational data from the power system, acquired via PMUs, are integrated with essential system parameters, including network topology, nodal characteristics, load weights, and microgrid output predictions. In this study, microgrid output is predicted using an LSTM neural network based on historical data and current system conditions, forecasting the future power generation of the microgrid. These predictions not only assist in island division but also provide the basis for prioritizing the restoration of critical loads, ensuring that key loads are considered first during restoration.

Initially, real-time operational data from the power system, acquired via PMUs, are integrated with essential system parameters, including network topology, nodal characteristics, load weights, and microgrid output predictions.

Next, BFS is used to traverse the grid, assigning unique identifiers to each node and branch, establishing an ordered network structure. This structure aids in island division, allowing the grid to be effectively partitioned into independent regions during a fault, facilitating recovery. Microgrid output predictions play a key role in island restoration, especially in prioritizing regions with critical loads to maximize their restoration times.

Then, the AMCGA is applied to optimize the island division schemes. Each chromosome in the population represents a potential partitioning solution, with the goal of maximizing critical load restoration time. Through selection, crossover, and mutation operations, the algorithm iteratively improves the restoration plan. Each generation is evaluated based on the restoration time, with longer restoration times corresponding to higher fitness. Multipoint crossover and mutation operations lead to gradual convergence, identifying the optimal restoration path. Maximizing the restoration time of critical loads is a primary focus during this process.

The entire process is optimized through the adaptive adjustment of crossover and mutation probability, enabling efficient optimization under different network configurations and fault scenarios, avoiding local optima. Ultimately, by integrating microgrid output prediction, island division, and genetic optimization, the power restoration process is optimized, improving restoration efficiency, ensuring priority restoration of critical loads, and maximizing their restoration times.

Compared with conventional GA, our proposed AMCGA demonstrates significant advantages in novelty, time complexity, and optimality. The core innovation of AMCGA lies in its adaptive multi-point crossover mechanism and dynamic parameter adjustment strategy, which dynamically tune crossover and mutation rates based on the population’s real-time fitness. This intelligent approach balances global exploration and local exploitation across different stages of the algorithm. This adaptiveness optimizes the convergence process, thereby reducing its time complexity by decreasing the number of iterations required to find a high-quality solution. Furthermore, this mechanism enables AMCGA to more effectively escape local optima, ensuring a higher quality of convergence for the final solution on key performance metrics. Consequently, it exhibits superior performance in power restoration applications that demand strict optimality.

Finally, to ensure compatibility with the MATLAB 2016a environment, the pseudocode for the algorithm to solve the power restoration strategy was written using appropriate MATLAB functions.

### 4.3. Comparative Analysis of AMCGA and Other Power Supply Restoration Algorithms

In addition to the AMCGA adopted in this paper, the main optimization algorithms for power system service restoration primarily include mathematical programming methods and artificial intelligence algorithms, represented by deep reinforcement learning. In theory, mathematical programming methods can find the optimal solution. However, this is often based on the linearization of key nonlinear constraints such as power flow, which not only introduces model errors but also causes a sharp increase in computational complexity as the scale of the power grid grows [31]. Although deep reinforcement learning shows potential in decision-making speed, its high offline training costs, reliance on massive amounts of data, and “black-box” nature pose practical challenges to its engineering applications. Table 1 provides a systematic comparison of these three types of algorithms across multiple key dimensions [32]. In summary, with its powerful global optimization capabilities and precise solutions for complex dynamic objectives, AMCGA emerges as the most appropriate and robust technical means for systematically seeking the optimal restoration strategy while satisfying all operational constraints of the power grid.

## 5. Results and Discussion

### 5.1. Description of Simulation Example

**Figure 2:**
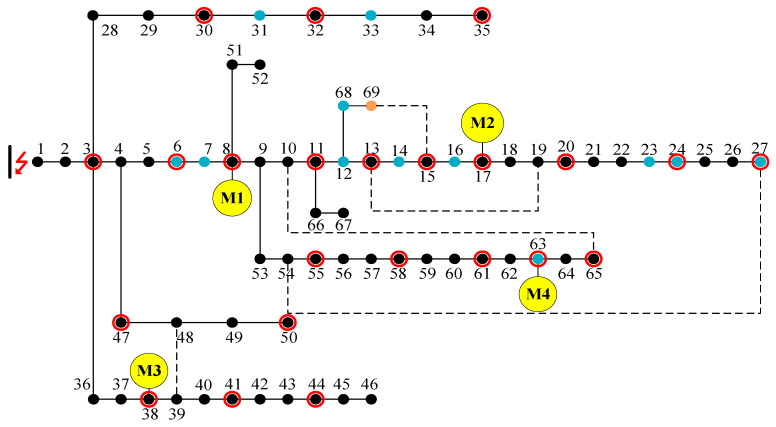
IEEE system designed in this study.

This study employs MATLAB 2016a as the simulation environment. Figure 2 illustrates the IEEE 69-node system implemented to validate the efficacy of the proposed power restoration strategy. The system incorporates four microgrids with diverse power ratings, positioned at nodes 8, 17, 38, and 63. The main grid outage scenario is scheduled from 00:00 to 23:00 on a clear day. Table 2 presents the comprehensive microgrid parameters, where red symbols indicate disconnected lines and yellow circles denote the respective microgrid locations. The dashed lines represent tie lines that function as auxiliary connections facilitating power supply within the system, enabling inter-regional power transfer and restoration between different nodes. To clarify the configuration scheme of PMU, we have marked the installation nodes with red circles in Figure 2. The nodes with PMU installed are as follows: 3, 6, 8 (M1), 11, 13, 15, 17 (M2), 20, 24, 27, 30, 32, 35, 38 (M3), 41, 44, 47, 50, 55, 58, 61, 63 (M4), 65. The primary load is represented by orange dots, and the secondary load is represented by blue dots. We use Algorithm 1 to solve the optimal power restoration strategy for this example.

**Algorithm 1:** Power Restoration Strategy Optimization1: Procedure: solve power restoration strategy(initial grid data, params) // where ‘initial grid data’ is collected from PMUs.2:  // Section 1: Initialization and Topological Analysis via BFS3:   Grid Model ← Initialize Grid Model (Initial grid data)4:5:   // Construct a Minimum Spanning Tree (MST) using Breadth-First Search6:   Start Node ← Find Main Power Source Node(Grid Model)7:   Queue Q ← [Start Node]8:   Set Visited Nodes V ← {Start Node}9:   Tree MST ← ∅10:    while Q is not empty do11:    u ← Dequeue(Q)12:    for each Branch S connected to u do13:     v ← Get other end of S14:     if v ∈ V then15:      Add v to V; Enqueue(Q, v); Add S to MST16:     end if17:    end for18:    end while19:20:    // Identify Chords and Form Fundamental Loops to Define the Search Space21:    Chords ← Calculate Set Difference(Grid Model.All Branches, MST)22:    Loop List ← ∅23:    for each Chord R in Chords do24:    Path ← Find Path in Tree(MST, R.Endpoint1, R.Endpoint2)25:    New Loop ← Path ∪ {R}26:    Add New Loop to Loop List27:    end for28:29:    // Synergy Point: The output of BFS (Loop List) now directly defines the chromosome structure for AMCGA.30:    // Chromosome Length = size of Loop List. Each gene corresponds to a loop.and gene values use decimal encoding.31:32:    // Section 2: Optimization via Adaptive Modified Genetic Algorithm (AMCGA)33:    Population ← Generate Random Initial Population(Params.Pop Size, Loop List)34:    for g ← 1 to $I_{\max}$ do35:    // Evaluate fitness for each individual solution36:    for each Chromosome in Population do37:     // Decode gene values into a set of open branches to form a new topology38:     Open Branches ← Decode Chromosome(Chromosome, Loop List)39:     Current Topology ← Calculate Set Difference(Grid Model.All Branches, Open Branches)40:     // Simulate grid operation over the specified duration to evaluate strategy effectiveness.41:     Total Score ← 042:     for time ← 0 to Simulation Hours do43:      DG Output ← Get DG Output44:      Restored Loads ← Calculate Restored Loads(Current Topology, DG Output)45:      Total Score ← Total Score +Calculate Weighted Score(Restored Loads)46:     end for47:     Fitness(Chromosome) ← Total Score48:    end for49:50:    // Evolution using Adaptive Multi-Point Crossover & Mutation51:    $P_{cross}=P_{c1}+P_{c2}*\frac{\left(I_{\max}-I\right)}{I_{\max}}$52:    $P_{mutation}=P_{m}*I_{\max}/\left(I_{\max}-m*I\right)$53:54:    Parents ← Select(Population, Fitness)55:    Offspring ← Multi Point Crossover(Parents, $P_{cross}$)56:    Offspring ← Mutate(Offspring, $P_{mutation}$)57:    Population ← Update Population(Population, Offspring)58:    end for59:60:    // Section 3: Formulate and Output the Final Strategy61:    Best Chromosome ← individual with highest Fitness in Population62:    Final Open Branches ← Decode Chromosome(Best Chromosome, Loop List)63:    Final Topology ← Calculate Set Difference(Grid Model.All Branches, Final Open Branches)64:    // The strategy includes the final network structure and could include other operational data.65:    Optimal Power Restoration Strategy ← Formulate Strategy(Final Topology, Grid Model)66:    return Optimal Power Restoration Strategy67:  end Procedure

**Figure 3:**
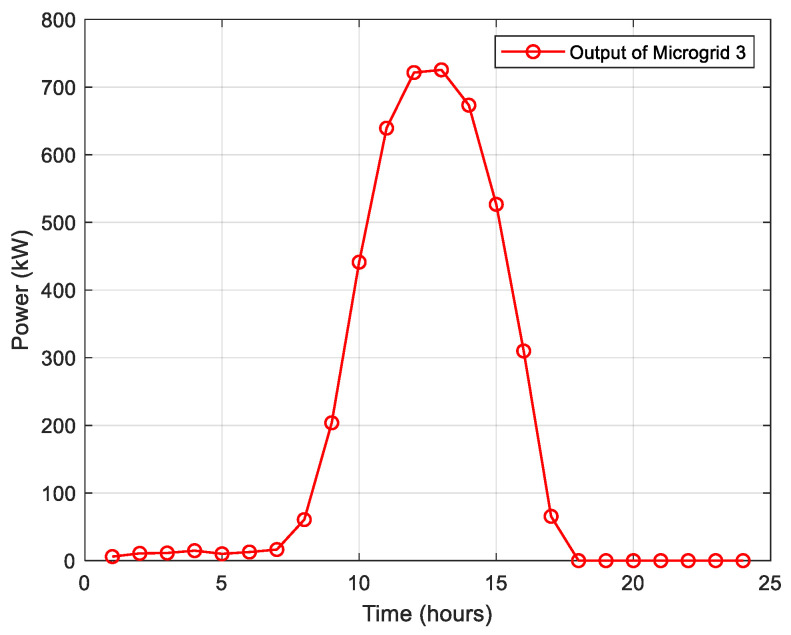
Predicted power generation profile of microgrid 3.

To illustrate the power generation characteristics, Figure 3 presents the forecasted power output profile of microgrid 3 throughout a complete 24 h operational period. This temporal distribution demonstrates the diurnal variation in available power resources during the restoration process.

**Figure 4:**
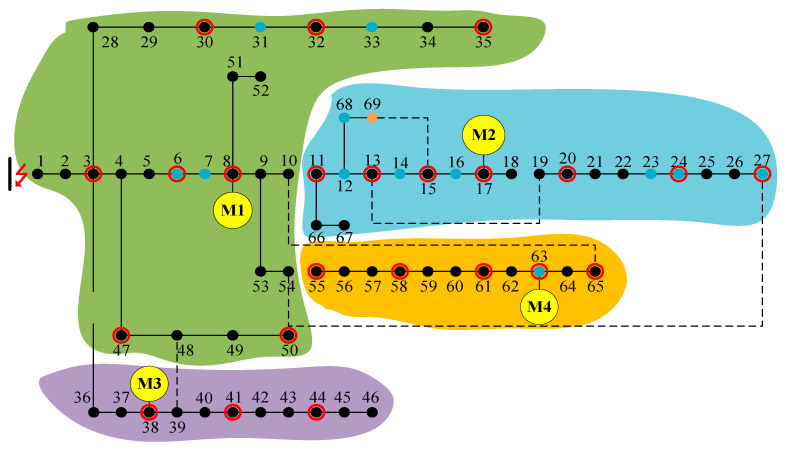
Island partitioning using the proposed recovery strategy.

The load types at each node within the power system are presented in Table 3, with varying levels of importance assigned to the loads at different nodes. To reflect this hierarchical importance in the optimization process, the weight coefficients for level I, II, and III loads are set at 100, 10, and 1, respectively. The parameters of the AMCGA are shown in Table 4. Figure 4 is the resulting topology of the power system following island partitioning. The distinct colored areas denote the individual islands formed by the proposed recovery strategy.

### 5.2. Results Analysis

This study first utilizes the BFS algorithm to identify network partitioning structures within the model, followed by the application of AMCGA to derive the optimal fault recovery strategy, ultimately obtaining the islanding configuration shown in Figure 4.

To quantitatively assess the continuity of critical load restoration and ensure an uninterrupted power supply to critical loads, the stability of critical load power restoration, denoted as δ, is introduced as a key performance indicator. This metric evaluates power supply fluctuations during the recovery process, with a smaller value indicating higher stability in the restoration of critical loads. It can be calculated using the following formula:

(10) $$\delta =\frac{1}{N}\cdot \frac{\sum_{i=1}^{69}\omega_{i}\cdot {\left(\mu_{i}-\bar{\mu }\right)}^{2}}{\sum_{i=1}^{69}\omega_{i}}$$

where N denotes the number of remaining time periods for the day, $\mu_{i}$ represents the recovery amount of the *i*-th critical node, $\bar{\mu }$ refers to the average recovery amount of the *i*-th critical node across all time periods, and $\omega_{i}$ represents the weight of the *i*-th node.

To comprehensively evaluate fault recovery performance under various optimization objectives, this study designs and compares three distinct strategies. Strategy A aims to maximize the restoration duration and continuity for critical loads. Strategy B prioritizes the total recovered load across the entire system. Strategy C seeks a balance by ensuring the restored power of critical loads while considering overall system benefits.

The differentiation of these strategies lies in the distinct weighting schemes applied to three load priority levels during two key phases of the genetic algorithm: the island partitioning process and the final fitness evaluation. Specifically, Strategy A utilizes a highly skewed weight set of (100, 10, 1) in both phases to grant absolute priority to critical loads. In contrast, Strategy B employs an equal weight set of (1, 1, 1) for all load levels, targeting only the total restored load. Strategy C adopts a hybrid approach, using the high-priority weights (100, 10, 1) during islanding to preferentially connect critical loads, but applying a more balanced set (1, 1, 1) in the final evaluation to balance the restoration of key nodes with overall network benefits.

As shown in Table 5, Strategy A excels in restoring critical loads, ensuring a primary load recovery duration of 6 h. This outperforms Strategy B, which restores critical loads for only 1 h, and Strategy C, which achieves 5 h. The extended recovery duration of Strategy A significantly improves power supply continuity for critical loads, enhancing overall power reliability. Additionally, Strategy A demonstrates superior stability, with a stability index of 1.1016, which is much lower than Strategy B’s 1.4084, indicating smoother recovery with fewer fluctuations. Strategy C, with a stability index of 1.1112, shows similar stability but falls short of Strategy A’s recovery duration. In terms of power restoration to critical loads, Strategy A delivers the highest power transfer of 1738.5 kW, ensuring a sustained and reliable supply to essential loads. This makes Strategy A the most effective approach for ensuring the stable restoration of critical loads.

Overall, Strategy A stands out for maximizing critical load recovery duration, providing a stable, smooth, and efficient recovery process, making it the most suitable option for power systems with strict requirements for critical load restoration.

**Figure 5:**
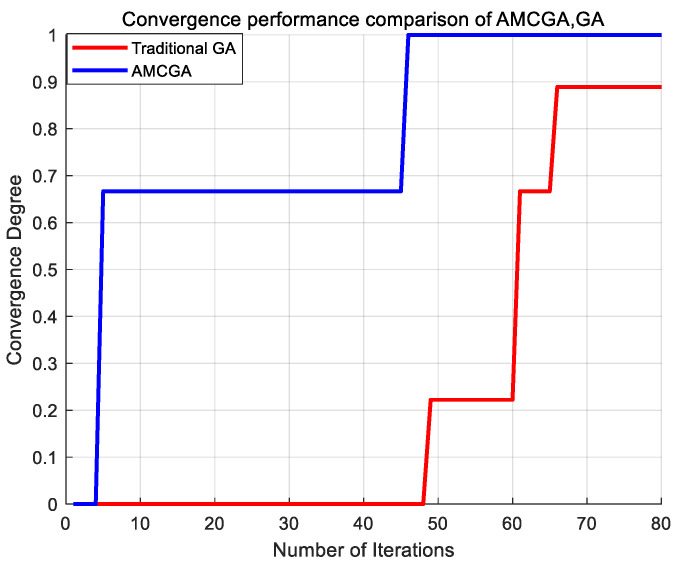
Convergence performance comparison of AMCGA and GA.

In order to demonstrate the superiority of the proposed AMCGA, the comparison results using AMCGA and conventional GA for the developed fault restoration model are shown in Table 6 and Figure 5.

**Figure 6:**
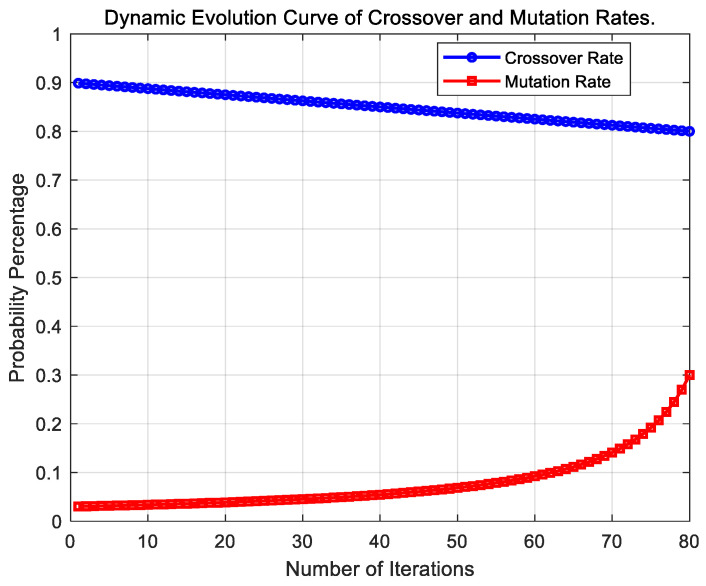
Dynamic evolution curve of crossover and mutation rates.

Figure 6 visually demonstrates how our AMCGA intelligently balances global exploration and local exploitation by adaptively adjusting its operator probabilities. In the initial stages, the algorithm employs a high crossover, low mutation strategy to facilitate a broad exploration of the solution space while protecting nascent high-quality genetic structures. As the iterations advance, the strategy smoothly transitions to maintaining a high crossover rate while significantly increasing the mutation rate. This shift enhances the algorithm’s capacity for refined local searching within the neighborhood of the optimum and provides the momentum to escape local optima, thereby ensuring efficient and robust convergence to the global solution.

This strategy directly corresponds to the rapid improvement in fitness values observed in our original best fitness evolution curve. Here, the fitness value serves as a direct measure of an individual solution’s quality, while the convergence degree is a normalized metric derived from fitness to assess the algorithm’s overall progress toward the optimal solution. Specifically, the convergence degree, as defined by Equation (11), maps the current best fitness to a [0, 1] scale, where a value approaching 1 signifies that the algorithm is nearing the theoretical optimum. Therefore, the rapid enhancement of fitness values directly translates to the steep ascent of the convergence curve.

The convergence degree is calculated using the following formula:

(11) $$\sigma =\frac{F_{current}-F_{\min}}{F_{\max}-F_{\min}}$$

where $F_{current}$ is the current fitness value, $F_{\min}$ is the worst fitness value, and $F_{\max}$ is the theoretical maximum fitness corresponding to the optimal solution.

As illustrated in Figure 5 and Table 4, the comparative analysis reveals significant performance differences between the optimization approaches. The tabulated results demonstrate that the conventional GA completes the full 80 iterations and achieves a convergence degree of 0.928, indicating a suboptimal solution. In contrast, AMCGA reaches a perfect convergence degree of 1 in merely 46 iterations-42.5% fewer than the maximum allowed. This performance differential underscores AMCGA’s superior optimization capabilities in maximizing critical load restoration duration, providing more reliable and sustained power supply to critical loads during system recovery. This establishes AMCGA as the more effective algorithmic approach for power restoration optimization in emergency scenarios.

When assessing the practical applicability of the proposed restoration strategy, it is imperative to consider its performance under real-world uncertainties. This model is primarily designed to address several key uncertainties:

To counter measurement uncertainty, the method utilizes an LSTM network to fuse historical and recent data, providing high-fidelity DG output forecasts for the critical recovery window of several hours post-fault, thereby significantly enhancing the quality of decision-making inputs. Regarding grid model uncertainty, the genetic algorithm is fundamentally engineered to actively manage post-fault topological changes, initiated from the faulted network state to dynamically compute an optimal reconfigured operational scheme. Finally, as the model concentrates on emergency restoration within a few hours, long-term planning uncertainties (e.g., annual load growth) are beyond its core scope.

**Remark** **1.** *In our current research, regarding operational uncertainties, we have not fully considered the uncertainty of load demand. Acknowledging this limitation, our future research will focus on incorporating stochastic or robust optimization techniques. Reference [2] effectively addresses the uncertainty in load demand using a stochastic optimization approach. Therefore, inspired by [2], we will explore methods for addressing the uncertainty of load demand through robust optimization in our future work.*

## 6. Conclusions

To ensure the prioritized and continuous restoration of critical loads during feeder outages, this paper proposes an electricity restoration strategy focusing on the maximization of critical load restoration duration. The strategy, supported by the AMCGA, aims to prioritize loads and ensure the continuous supply of power to critical loads during the restoration process, thereby maximizing their restoration duration. The conclusions are as follows:

(1) The proposed power restoration model successfully prioritizes the restoration of critical loads and maximizes their restoration duration. The core objective of the model is to ensure that critical loads remain in the priority restoration state throughout the recovery process, thus extending their restoration time to the greatest extent and significantly improving the overall effectiveness and timeliness of critical load recovery.
(2) The introduction of AMCGA, through dynamic adjustment of crossover and mutation rates, expands the solution space and accelerates the convergence process. This algorithm significantly enhances the solution efficiency of the restoration strategy, enabling the rapid identification of the optimal power restoration strategy and ensuring the maximization of critical load restoration duration.
(3) The integration of microgrid-based islanding and load priority allocation ensures that, during the restoration process, critical loads receive priority and a continuous power supply. This strategy not only enhances the stability of the restoration process but also further extends the restoration duration of critical loads, ensuring the sustained recovery and stable power supply for critical loads.

Despite its promising performance, the proposed strategy has several limitations that provide directions for future research. While the AMCGA demonstrates effectiveness in medium-sized networks, such as the IEEE 69-bus system, its scalability in larger-scale, more complex systems remains to be further validated. Furthermore, the algorithm’s effectiveness has not been tested with real-world distribution network data. Future work will focus on addressing these limitations to enhance the practical feasibility of the approach. We plan to test the method on larger and more formal distribution systems to validate its performance and computational efficiency in more complex scenarios. The engineering scenarios presented in [31], which utilize real-world distribution network data, provide valuable references and practical environments for the future validation of our proposed strategy. We will seek further cooperation with the authors of this literature and request their dataset to validate our method.

## Figures and Tables

**Table 1:** Strategy comparison.

Algorithm	AMCGA	Mathematical Programming Methods	Deep Reinforcement Learning Methods
Solution Convergence	Can obtain global optimum/near-optimum solution	Can obtain global optimum under a linear model	Cannot guarantee the optimality of a single decision
Model Adaptability	Very strong; can flexibly handle nonlinear models	Weaker; requires model linearization, difficult to handle non-homogeneous constraints	Moderate; hard constraints need to be handled during the learning process
Solution Speed	Relatively slow	Slow; increases with problem size	Very fast, but offline learning is time-consuming
Algorithm Dependency	Relies on parameter tuning	Relies on precise mathematical models and high-precision solvers	Relies on massive training data and reward function design
Core Advantage	A high balance between global search and model adaptability	Theoretically complete; the optimality of the solution is guaranteed	Fast decision-making, with learning and adaptive capabilities
Applicable Scenarios	Complex scenarios with high requirements for solution quality	Small-scale linear programming problem scenarios	Scenarios with massive amounts of training data

**Table 2:** Parameters of the microgrid.

Microgrid ID	Microgrid1 (M1)	Microgrid2 (M2)	Microgrid3 (M3)	Microgrid4 (M4)
Connection Node	8	17	38	63
Rated Power/kW	500	500	1000	750
DG Type Included	Photovoltaics	Photovoltaics	Photovoltaics	Photovoltaics

**Table 3:** Node load types.

Frequency Regulation Performance	Load Weight	Corresponding Nodes
Primary Load	100	69 (orange-yellow nodes in Figure 2)
Secondary Load	10	6 7 12 14 16 23 24 27 31 33 63 68 (blue nodes in Figure 2)
Tertiary Load	1	All remaining nodes (black nodes in Figure 2)

**Table 4:** Parameters of the AMCGA.

Parameter	Value
Population size	30
Number of generations	80
Initial crossover rate	0.8
Initial mutation rate	0.03
Crossover rate adjustment coefficient	0.1
Mutation coefficient	0.9
Elite individuals count	15

**Table 5:** Critical load recovery metrics and power transfer indicators under different strategies.

Recovery Strategy	Strategy A	Strategy B	Strategy C
Primary Load Recovery Duration on the Same Day After Fault/h	6	1	5
Total Secondary Load Recovery Duration on the Same Day After Fault/h	97	92	92
Critical Load Power Restoration Stability	1.1016	1.4084	1.1112
Total Power Transfer of Critical Loads/kW	1738.5	1621.6	1281.5

**Table 6:** Comparison of performance metrics for different algorithms.

Comparison of Algorithms	GA	AMCGA
Number of Iterations for Convergence	80	46
Maximum Convergence Degree	0.928	1

## Data Availability

The original contributions presented in this study are included in the article. Further inquiries can be directed to the corresponding author.
